# Genotoxic capacity of Cd/Se semiconductor quantum dots with differing surface chemistries

**DOI:** 10.1093/mutage/gev061

**Published:** 2015-08-14

**Authors:** Bella B. Manshian, Stefaan J. Soenen, Andy Brown, Nicole Hondow, John Wills, Gareth J. S. Jenkins, Shareen H. Doak

**Affiliations:** ^1^Institute of Life Science, College of Medicine, Swansea University, Singleton Park, Swansea SA2 8PP, UK,; ^2^Biomedical NMR Unit-MoSAIC, Department of Medicine, KU Leuven, B-3000 Leuven, Belgium and; ^3^Institute for Materials Research, SCaPE, University of Leeds, Leeds LS2 9JT, UK

## Abstract

Quantum dots (QD) have unique electronic and optical properties promoting biotechnological advances. However, our understanding of the toxicological structure–activity relationships remains limited. This study aimed to determine the biological impact of varying nanomaterial surface chemistry by assessing the interaction of QD with either a negative (carboxyl), neutral (hexadecylamine; HDA) or positive (amine) polymer coating with human lymphoblastoid TK6 cells. Following QD physico-chemical characterisation, cellular uptake was quantified by optical and electron microscopy. Cytotoxicity was evaluated and genotoxicity was characterised using the micronucleus assay (gross chromosomal damage) and the HPRT forward mutation assay (point mutagenicity). Cellular damage mechanisms were also explored, focusing on oxidative stress and mitochondrial damage. Cell uptake, cytotoxicity and genotoxicity were found to be dependent on QD surface chemistry. Carboxyl-QD demonstrated the smallest agglomerate size and greatest cellular uptake, which correlated with a dose dependent increase in cytotoxicity and genotoxicity. Amine-QD induced minimal cellular damage, while HDA-QD promoted substantial induction of cell death and genotoxicity. However, HDA-QD were not internalised by the cells and the damage they caused was most likely due to free cadmium release caused by QD dissolution. Oxidative stress and induced mitochondrial reactive oxygen species were only partially associated with cytotoxicity and genotoxicity induced by the QD, hence were not the only mechanisms of importance. Colloidal stability, nanoparticle (NP) surface chemistry, cellular uptake levels and the intrinsic characteristics of the NPs are therefore critical parameters impacting genotoxicity induced by QD.

## Introduction

The nanotechnology industry is rapidly expanding with nanomaterials destined to be incorporated into various electrical applications and consumer products, or used for an array of applications such as biomolecular monitoring, medical imaging and therapeutic interventions (1,2). Indeed large investments are being made by many industrial and government organisations towards the development of these technologies with upper estimates of market value of nearly $64.2 billion in 2019 (3). Semiconductor nanoparticulate quantum dots (QD) are one such type of nanomaterial that have shown great promise in the field of imaging and electronics (4). When optically excited (typically in the UV range) QD emit photons in the visible range and at a wavelength directly dependent on composition and size (5). The high brightness, low photo-bleaching, narrow emission spectra combined with a large Stoke’s shift, and size-dependent emission maxima render these particles ideal tools for advanced fluorescence imaging. As such QD are likely to be particularly beneficial in long-term bio-imaging or for the visualisation of rare cellular events (6,7). The ability to excite with a single wavelength and then detect differences in emission (e.g. based upon size) further opens up opportunities in multiplexing; where several biological events can be tagged and investigated simultaneously (8–10).

For a multitude of nanomaterials, significant degrees of toxicity have already been shown by *in vitro* and *in vivo* studies (11–13). Widespread manufacture and use of QD comes with the risk of increased human and environmental exposure to, in some cases, significant amounts of these particles (14). When used in biomedical applications these materials will likely be introduced into patients, however, disassembly of consumer products containing QD may also result in their release into the environment at high local doses, where they might accumulate and degrade (15,16). Our current knowledge of the potential health effects of exposure to QD is mainly derived from acute cytotoxicity studies, and the data generated suggest that QD may exert adverse effects in the skin (17), lungs (18,19), gastrointestinal tract (20) and other tissues (21,22). Yet, the debate on the potential toxicity of QD still persists; for instance, no toxicity could be found in a pilot study on non-human primates (23). It has been suggested that as QD are not excreted efficiently, this could lead to potential long term (chronic) exposure problems (24). Furthermore, several reports have suggested that correlating *in vitro* to *in vivo* findings are problematic and that more factors, such as the effect of nanoparticle (NP) dosing should be taken into account (25,26). It is also becoming increasingly apparent that any observed biological findings must be carefully correlated with the physicochemical properties of the QD, as the many variations in chemical composition, structure, coating agents and sizes make it very hard to derive any general conclusion on toxicity (13,23).

One factor that has received little attention to date is the intrinsic genotoxicity of QD, which is of some concern as the most common, cadmium containing QD, may have genotoxic or carcinogenic effects if cadmium is released into cells or the body due to QD breakdown in acidic environments such as those found in lysosomes (22,27,28). Cadmium metal is known to be very toxic to humans and in several countries its use is prohibited in certain products. Cellular or tissue uptake of heavy metals such as cadmium can lead to the generation of reactive oxygen species (ROS) that can result in oxidative stress induced DNA damage (29–31). Although some studies have shown that QD can cause cytotoxic damage at specific doses, whether this is true for all cell types, all QD variants and what the underlying modes of action are remains areas of limited understanding. There are a small number of studies indicating that QD do have some capacity for inducing DNA damage (22,32–34). However, many such reports frequently only consider a single type of QD or cell lines are utilised that are not wholly suitable for genotoxicity testing (e.g. due to high background genetic instability or cancer-derived cell lines are employed that may be more resistant or sensitive to DNA damage). The aim of this study was therefore to investigate the interaction and potential (geno)toxicity of QD with a series of different surface modifications in a cell line well-established for genotoxicity assessment, human lymphoblastoid TK6 cells (35). The use of three QDs with similar chemical composition but with different surface functionalities (negative, neutral and positive chemistries) enabled us to address the role of QD surface functionalisation as a main determinant in the cellular uptake and resulting genotoxicity of the NP. Multiple endpoints were investigated including cytotoxicity, mutagenicity and chromosomal damage, coupled to an exploration of the possible mechanistic role for oxidative stress.

## Materials and methods

### QD nanoparticles

CdSe/ZnS core/shell fluorescent nanocrystals with -amine (Cytodiagnostics, Canada), -carboxyl (COOH, Invitrogen, UK), and -hexadecylamine, HDA (Sigma–Aldrich, UK) functional ligands attached to the surface were used. The emission maxima of each QD were 585, 590 and 665nm for the carboxyl-, HDA- and amine-QD, respectively. The QD selected had the same primary core, with an average diameter range of 4–10nm including both the CdSe core and ZnS shell according to the manufacturer’s notes. Prior to cell exposure, carboxyl- and amine-QD were suspended in water, while the HDA-QD were re-suspended in 1% dimethyl sulfoxide (DMSO) in phosphate buffer saline (PBS) solution.

### Physico-chemical characterisation studies

Primary QD diameter, morphology and crystallinity were analysed by transition electron microscopy (TEM) by drop casting the as-purchased QD suspensions onto copper grids coated with a holey carbon support films (Agar Scientific Ltd). Additionally, the composition of the NPs was evaluated by energy dispersive X-ray elemental analysis (EDX) using an Oxford Instruments INCA 350 EDX system with an 80mm^2^ X-Max SDD detector.

The agglomeration of the QD was measured by dynamic light scattering (DLS) to determine their hydrodynamic diameters and zeta potentials using a Malvern 4700 system (Malvern instruments Limited, UK). The QD were suspended at 15nM in water or RPMI-1640 medium with and without 1% horse serum (HS), sonicated for 30 s and analysed at 37°C to mimic cellular exposure conditions. Results are presented as the average of 30 readings (10 readings per replicate). Polydisperion index (PDI) values were recorded for all the readings.

### Cell culture

Human lymphoblastoid-B TK6 suspension cell lines were purchased from ATCC (ATCC Cell lines Service, USA) and were maintained in 75cm^2^ flasks at a concentration of 1.5×10^5^ cells/ml. Cells were cultured in Rosewell Park Memorial Institute (RPMI-1640) medium supplemented with 1% 2mM L-glutamine (Gibco®, UK), and 10% HS (Gibco®, UK). Cells were maintained in an incubator set to an atmosphere of 37°C and 5% CO_2_. For all the experiments cells were seeded at 1.5×10^5^ cells/ml in culture medium containing reduced (1%) serum and allowed to settle overnight prior to treatment with QD for 18h (1-cell cycle). The reduced serum concentration selected was based on optimization studies to identify the lowest serum content that could be applied for the experimental duration without altering cell growth parameters (data not presented).

### pH effect on QD breakdown

QD stability under different biological pH levels were determined by preparing different solutions containing 1% HS mixed with PBS and the pH was adjusted to 7.4, 5.5 and 4.5. QD were prepared at 2.5, 7.5, and 10nM concentrations in 100 µl total volume accompanied with negative control preparations. Samples were aliquoted into dark 96-well tissue culture plates (Greiner Bio One BVBA, Belgium) in triplicate. Fluorescence measurements were taken using the Omega multiwell plate reader (BMG Labtech, Belgium) immediately following the addition of the QD (Day 0) and on the following days until Day 4 post preparation.

### Cellular uptake evaluation

For ImageStream, confocal microscopy and TEM analyses TK6 cells were seeded overnight at 1.5×10^5^ cells/ml in media containing 1% HS. The following day cells were exposed to 15nM of each QD in media containing reduced (1%) serum for 18h. Particle suspensions were sonicated for 30 s before introduction to the cell culture. At the end of the incubation period cells were washed twice with sterile PBS and harvested by centrifugation.

### ImageStream flow cytometry

For image based flow cytometry, treated and harvested cells were fixed for 30min at room temperature with FACS fixative solution (BD Biosciences, UK). Fluorescent images of 5000 cells were acquired for each sample using the Image Stream flow cytometer (Amnis Corporation) with excitation at 488nm for all samples and collection at 488nm (carboxyl- and HDA-QD) or 633nm (amine-QD). All experiments were conducted in duplicate and data were analysed using the IDEAS software (Amnis Corporation). This analysis software allowed the selection of focused-single cells which verifies that the QD fluorescence quantified is generated only from QD inside the cell and not from particles adhering to the external side of the cellular membrane.

### Confocal microscopy

For further confirmation of QD internalization by TK6 cells, cellular uptake was investigated with confocal microscopy. Following the exposure period cells were removed and seeded at 4×10^4^ cells/well into 35mm glass bottom Lab-Tek chamber slides (Thermo Scientific, UK) coated with Poly-l-lysine (Sigma−Aldrich, UK). The cells were allowed 4h to settle and attach at 37°C in a humidified atmosphere, then the media was removed, the cells carefully washed three times with sterile PBS and fixed with FACS fix (BD Biosciences, UK) for 30min at room temperature. Cells were washed once more with PBS and 1% Hoechst nuclear counterstain was added to each well for 2min at room temperature. The stain was removed by washing with PBS and fresh PBS was added to each well followed by visualisation under an inverted Zeiss LSM 710 laser scanning confocal microscope (Carl Zeiss, Inc., UK). Laser excitation was *via* a 25-mW bulb and 488, 561 and 633nm lasers were used for the different QD. The localisation of the QD in the intracellular environment was confirmed by 3D imaging facilitated by the collection of a series of images along the *z*-axis. Depending on the cell size, the resultant *z*-stack comprised of approximately 25–30 slices.

### Transmission electron microscopy

Exposed cells were centrifuged to collect a cell pellet which was fixed with 2.5% phosphate buffered (pH 7.3) glutaraldehyde (2h) prior to post-fixation in 1% Millonig’s buffered (pH 7.3) osmium tetroxide (120min). Samples were then dehydrated through an ethanol series (10min each, 10%, 70%, 100%), then transferred to 100% propylene oxide and resin embedded. Ultrathin-sections (70nm) were then cut for each cell pellet using a Leica EM-UC7 microtome and Diatome Ultra 45° diamond knife. Grids were carbon coated and images captured using a FEI Tecnai TF20 FEG-TEM operating at 200kV coupled with a Gatan Orius SC600A camera and an Oxford Instruments INCA 350 EDX system with an 80mm^2^ X-Max SDD detector. For each cell treatment assessed, >10 cell sections were analysed.

### Relative population doubling

Cell viability was measured using the relative population doubling (RPD) assay where 1.5×10^5^ cells/ml were seeded overnight in T25 flasks in 10ml culture medium containing 1% HS. The following day cells were treated with QD suspended in PBS (amine- and carboxyl-QD) or 1% DMSO (HDA-QD) and for sonicated 30 s prior to cellular exposure. All experiments were conducted in duplicate. Following 18h exposure, cells were washed three times with sterile PBS and incubated in fresh media containing full serum for another 18h to allow for cellular repair. Finally cells were harvested and counted. Cytotoxicity was determined according to the population doubling of each treated sample compared with the initial cell count and relative to the control as follows:

$$\text{RPD}=\frac{\left(\text{No}.\text{ of population doublings in treated cultures}\right)}{\left(\text{No}.\text{ of population doublings in control cultures}\right)}\times 100$$

where:

$$\begin{array}{l} \text{Population Doubling}=[\log(\text{Post-treatment cell number}\\ ÷\text{ Initial cell number})]÷\text{log}2 \end{array}$$

### Automated cytokinesis blocked micronucleus assay

Cells were seeded overnight at 1.5×10^5^ cells/ml in 10ml reduced serum containing media. The following day cells were treated with QD, negative and positive (mitomycin-C (MMC) 0.01 µg/ml) controls in the reduced media. Following 18h exposure, cells were washed and re-incubated in culture media supplemented with 3 μg/ml cytochalasin B for 18h after which cells were harvested and fixed in hypotonic treatment: KCl 0.56% and centrifuged immediately for 10min followed by 10min incubation in Fixative 1; methanol: acetic acid: 0.9% NaCl (5:1:6 parts). Finally, the harvested cell pellet was fixed for 10min in Fixative 2; methanol: acetic acid (5:1 parts), and centrifuged at 4°C. The wash step was repeated four times. Fixed cells were incubated overnight at 4°C in the second fixative. The Metafer automated system from Metasystems was then used to quantify micronuclei in binucleated cells. Prior to image acquisition, cells were dropped onto slides and stained with 4′,6-diamidino-2-phenylindole (DAPI) for 10min in the dark. Slides were then mounted onto the Metafer stage and the number of micronuclei determined in binucleated cells according to a set of pre-optimised parameters. Duplicate samples were prepared per dose and in total 6000 cells were analyzed from both replicates.

### HPRT forward mutation assay

The Hypoxanthine-guanine phosphoribosyl transferase (*hprt*) forward mutation assay was performed to quantify mutagenicity. Background *hprt* gene mutants were removed from TK6 cells by growing them for 3 days in media containing aminopterin (2×10^−4^ M hypoxanthine / 8×10^−7^ M aminopterin / 3.5×10^−5^ M thymidine). Cells were then seeded at 5×10^5^ cells/ml in 10ml reduced serum containing media and were exposed to QD, negative and positive controls. Following 18h incubation cells were washed three times with sterile PBS, re-suspended in full serum containing media and cultured for 13 days to allow the development of mutations in both strands. During the incubation period, cells were maintained at a concentration of 1.25×10^5^ cells/ml to avoid overgrowth. On Day 14, cells were harvested by centrifugation, re-suspended at 4×10^4^ cells/ml in fresh media supplemented with 0.6 µg/ml 6-thioguanine and were placed into 5×96-well plates (4000 cells/well) for the detection of mutation frequency (MF). Additionally, five plates without selection were prepared containing 200 cells/well to determine the plating efficiency. Experiments were performed in triplicate and were accompanied by negative diluent-only control and positive (N-nitroso-N-methylurea (MNU) at 0.04 µg/ml) controls. At the end of the experiments colony formation was scored to determine the MF.

### ROS and mitochondrial ROS evaluation

For both ROS and mitochondrial ROS (mito-ROS) experiments, 2×10^5^ cells/ml were seeded in black 96 well plates (Greiner Bio One, UK) and allowed to settle overnight. Cells were exposed to QD for 4, 8 and 18h, washed three times with PBS and incubated for 30min in full culture media supplemented with 10 µM 5-(and-6)-chloromethyl-2′,7′-dichlorodihydrofluorescein diacetate, acetyl ester (CM-H_2_DCFDA; Molecular Probes, Invitrogen, UK) for ROS or 20 μM JC-10 (Enzo Life Sciences, UK) for mito-ROS assessment. Cells were washed twice with PBS and fluorescence readings were taken in an Omega microplate reader (BMG Labtech, UK) at 480 and 520nm excitation and 540 and 590nm emission for ROS and mito-ROS analyses respectively, according to the manufacturer’s instructions. For mito-ROS experiments, results were expressed as the ratio of damaged over healthy mitochondria (green/red). For both ROS and mito-ROS experiments, data were expressed as relative to the control level. All experiments were conducted in triplicate and were accompanied with negative diluent only controls and positive control treatments of 0.33M (1%) H_2_O_2_ for 2h prior to chemical staining.

### Statistical analysis

All data are expressed as the mean ± standard deviation (SD). Image Stream results are presented as relative fluorescence compared with the untreated control cells and are expressed as the mean ± standard error of the mean. Significance in the binucleated micronucleus frequency data was evaluated using the Fisher’s exact test. The *hprt* mutant frequency data was first assessed for a normal distribution using the Shapiro–Wilk test. When normality was demonstrated, Levene’s test for equality of variances was performed to ensure the assumptions for parametric tests were not violated. The data was then logarithmically transformed and analysed with a one-way ANOVA. Data generated by the ROS and mito-ROS experiments were also analysed using the one-way ANOVA statistical method. For all statistical analysis, *P* > 0.05 was considered significant.

## Results

### Characterisation of QD physicochemical properties

**Figure 1.**
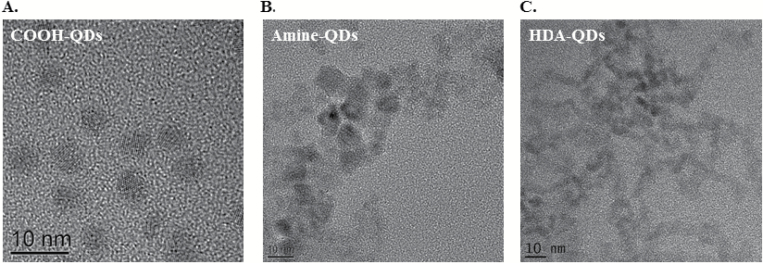
TEM images of test QD. (**A**) Carboxyl-QD, (**B**) amine-QD, (**C**) HDA-QD. All QD were prepared for imaging in their as-purchased solutions.

Assessment of primary particle characteristics was obtained by TEM imaging. The QD were analysed by drop-casting from the solutions that they were supplied in onto thin carbon films. As illustrated in Figure 1, amine-QD were generally crystalline in structure and ranged between 3 and 10nm in diameter, while carboxyl-QD were crystalline and had a diameter of 4–5nm. The HDA-QD, on the other hand, were crystalline and elliptical in shape, approximately 4nm × 8nm in dimension (Figure 1). EDX analysis demonstrated that the QD contained no impurities and only consisted of Cd, Se, Zn and S.

Agglomeration under experimental conditions was evaluated by DLS. Upon analysing the hydrodynamic diameter of the QD it was found that there was a wide distribution of agglomerate sizes in water and in media containing 1% HS (Table 1). In media with 1% serum, carboxyl-QD had the smallest median agglomerate size followed by the HDA-QD and then the amine-QD. Zeta potential measurements were used to establish QD surface charge; this is most representative of the particle surface charge when measured in water as the evaluation is not compromised proteins in the media or by the formation of a protein corona on the dots. In water, amine-QD demonstrated a slight positive zeta-potential of 8.7 mV, while the carboxyl-QD had a strong negative zeta potential of −30.4 mV. The HDA-QD had a zeta potential of −18.5 mV. In media these values all become negative, presumably because of protein attachment to the surfaces.

**Table 1.** Hydrodynamic diameter and zeta potential of 15nM carboxyl-, amine- and HDA-QD dispersed in filtered water or RPMI medium containing 1% HS

	Carboxyl-QD		Amine-QD		HDA-QD	
	Water	1% RPMI	Water	1% RPMI	Water	1% RPMI
Size distribution (nm)	91–1106	4.8–712	295–1106	164–955	37–458	50–955
Median diameter (nm)	255	13.54	615.1	342	141.8	164.2
PDI	0.4	1.00	0.789	0.395	0.372	0.538
Zeta potential	−30.4	−9.64	+8.71	−4.79	−18.5	−7.58

All samples were analysed at 37°C.

### pH effect on QD stability

**Figure 2.**
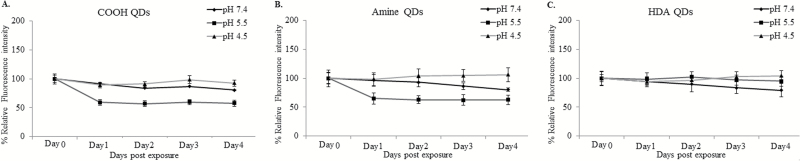
Graphs representing the percent relative fluorescence intensity detected from 10nM (**A**) carboxyl-, (**B**) amine- or (**C**) HDA-QD in 1% HS adjusted to varying pH conditions (7.4, 5.5, 4.5). Results of Days 2, 3 and 4 are compared with those of Day 0 and percent values presented as mean ± standard deviation (*n* = 3).

Exposing QD to three different biological pH levels (7.4, 5.5 and 4.5) revealed that carboxyl- and amine-QD started to degrade by Day 2, particularly in the pH 5.5 environment (Figure 2). In contrast, HDA-QD degradation was slower with no notable effects until Day 4 in pH 7.4.

### TK6 cell uptake of QD

Intracellular internalisation and localisation of QD were confirmed using three image based methodologies; the semi-quantitative image based flow cytometry (ImageStream analysis), confocal microscopy and TEM. These methodologies allowed visualisation of the NPs at a range of spatial resolutions, coupled to robust quantification of uptake with the high throughput ImageStream system.

**Figure 3.**
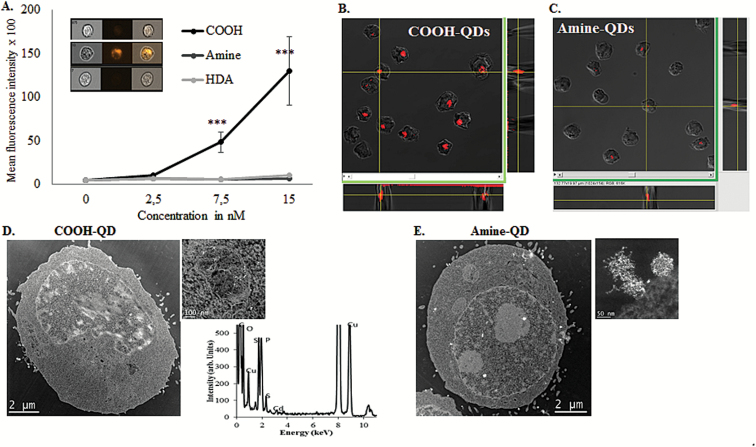
Evaluation of QD uptake by TK6 cells. (**A**) Graph representing the mean fluorescence intensity detected during Imagestream flow cytometry of TK6 cells exposed to the QD. (**B**, **C**) Orthogonal view presentation of *z*-stacks acquired during confocal microscopy. Greater uptake of carboxyl-QD as compared to and amine-QD (red) can be seen in the images presented (acquired images did not reveal any cells with HDA-QD uptake and are therefore not shown). (**D**, **E**) TEM images illustrating uptake of carboxyl- and amine-QD. Small images represent magnified regions of the larger whole cell view. Carboxyl-QD can be seen inside endosomal compartments within cells while amine-QD appear mostly to adhere to the surface of the cells. Results are presented as mean ± standard deviation (*n* = 3). Significance is indicated with ***P ≤ 0.001.

ImageStream analysis of QD cell uptake was utilised as it enables the high-throughput analysis of thousands of cells for fluorescence response (representing QD internalisation), and thus is a powerful tool for quantitative uptake evaluation. ImageStream outputs demonstrated that the most significant levels of QD uptake by TK6 cells were seen when they were exposed to carboxyl-QD (Figure 3A). In contrast, uptake of both amine- and HDA-QD was substantially more limited. At the top dose of 15nM there was a slight increase in the fluorescence response of TK6 cells exposed to both amine- and HDA-QD, suggesting low-level uptake, but this did not achieve significance.

The fluorescent properties of QD make them amenable to imaging by confocal microscopy, to provide a higher resolution but less quantitative view of NP internalisation. Thus, uptake of all three QD into TK6 cells was assessed by confocal microscopy. Z-stack images were collected in order to provide 3D data which, along with orthogonal analysis, also clearly demonstrated that carboxyl- and amine-QD were internalised by the cells (Figure 3B and C). Despite the ability to analyse greater cell numbers with confocal microscopy, again no evidence of HDA-QD uptake could be found.

TEM was utilised to establish sub-cellular localization of the QD. High angle annular dark field (HAADF) scanning TEM (STEM) was utilised in the examination of the thin sections, as the atomic number contrast allows for easy identification of high atomic number containing features, such as the cadmium-containing QD. Uptake of carboxyl-QD in the TK6 cells was clearly observed (Figure 3D). Nanoparticles were largely located within membrane bound vesicles, but occasionally small agglomerates of the QD were noted to be free within the cytoplasm. Amine-QD were only imaged co-localised to the outside of the cell membranes (Figure 3E). In contrast, no HDA-QD were detected in any of the examined cells. It is important to note that this does not indicate cell uptake of HDA-QD was absent, however, the comparatively lower uptake of these QD meant that a sufficiently large enough number of ultra-thin cell sections was impractical to assess by TEM to in order to locate these NPs.

### Cell viability and chromosomal damage induction

**Figure 4.**
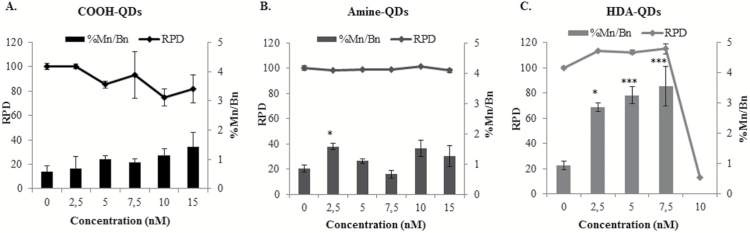
Cytotoxicity and chromosomal damage induced by QD. (**A**) Carboxyl-QD, (**B**) amine-QD, (**C**) HDA-QD exposed to TK6 cells for 18h. Graphs represent the RPD results and frequency of micronucleus induction in binucleated cells. Data are presented as mean ± standard deviation (*n* = 2). Significance is indicated with *P ≤ 0.05 and ***P ≤ 0.001.

RPD analysis revealed no notable cytotoxic effects from exposure of TK6 cells to amine-QD up to 15nM (Figure 4). Carboxyl-QD did induce a dose-dependent decrease in cell viability, but this did not reach significance. In contrast, a highly significant decrease (>50% RPD) in cell viability was noted following the exposure of TK6 cells to HDA-QD at ≥10nM (Figure 4C).

These analyses were accompanied with parallel experiments investigating the generation of micronuclei in TK6 cells exposed to the three QD. In total, 6000 cells (2000 cells per replicate) were analysed per dose, using the Metafer automated system with a positive control of MMC 0.01 µg/ml, which resulted in 3.08±0.44% micronuclei. Although exposure to carboxyl-QD resulted in a dose dependent increase in micronuclei induction, this did not reach significance. Amine-QD also did not induce notable increases in micronuclei with exception of the lowest dose of 2.5nM, which resulted in a significant near-doubling in micronucleus frequency (Figure 4). In contrast, HDA-QD generated a significant dose-dependent increase in gross chromosomal damage up to 7.5nM (Figure 4C). No analyses were conducted for the HDA-QD at 10 or 15nM concentrations due to the presence of more than 50% cell death.

### Induction of mutagenicity by QD

**Figure 5.**
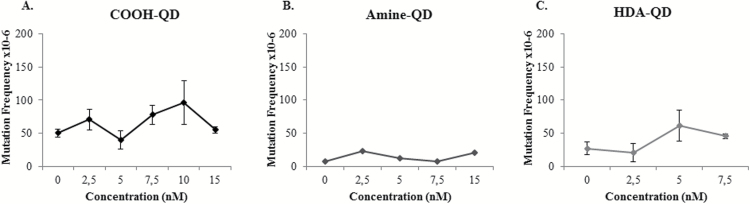
Point mutagenicity induced by QD. (**A**) Carboxyl-QD, (**B**) amine-QD, (**C**) HDA-QD exposed to TK6 cells for 18h. Results are presented as mean ± standard deviation (*n* = 3). Significance is indicated with *P ≤0.05 and **P ≤ 0.005.

The mammalian *hprt* forward mutation assay was applied to quantify cells containing point mutations following QD exposure, where the positive control induced a MF of 197±70×10^−6^. Carboxyl-QD resulted in a dose-dependent increase of point mutations up to 10nM where an approximate 2-fold increase in MF was observed (Figure 5A) compared with the control levels. This increase in MF was however not maintained at the highest 15nM dose and those elevations in MF observed at the lower doses did not reach significance. Similarly, an increase in MF was observed in TK6 cells following exposure to 5 and 7.5nM concentrations of HDA-QD where approximately 3- and 2-fold increase in MF were recorded respectively, but this again did not reach significance (Figure 5C). In contrast, no noticeable mutagenicity was caused by exposure to the amine-QD (Figure 5B).

### ROS and mito-ROS assessment

**Figure 6.**
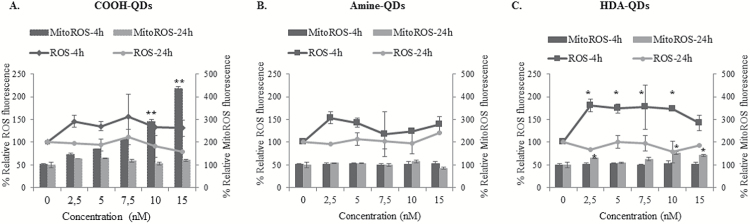
Oxidative stress induced following exposure to QD. (**A**) Carboxyl-QD, (**B**) amine-QD, (**C**) HDA-QD exposed to TK6 cells for 4 or 18h. Line graphs represent ROS, while bar graphs represent mito-ROS. Results are presented as mean ± standard deviation (*n* = 3). Significance is indicated with *P ≤ 0.05 and **P ≤ 0.005.

Having observed significant pockets of genotoxicity following exposure to QD, further experiments were conducted to evaluate the association of oxidative stress in response to QD of different surface chemistries. The production of both ROS and any changes to the mitochondrial membrane potential (Δψ_m_) were examined following 4 and 18h exposure periods. The 4h time point was selected to ensure the detection of early oxidative species; while the later time point represents the cell state at the end of the exposure period. Carboxyl- and amine-QD did not result in the significant induction of ROS in TK6 cells following either 4 or 18h exposure (Figure 6). However, HDA-QD demonstrated a clear increase in ROS levels, at all the tested concentrations up to 10nM, following treatment for 4h (Figure 6C dark line). These ROS elevations were diminished after 18h exposure.

Although carboxyl-QD did not increase ROS, they did induce significant increases in mito-ROS at 4h in a dose-dependent manner, with approximately 3- and 4-fold increases at 10 and 15nM, respectively compared with the control (Figure 6A). By the 18h time point, normal mito-ROS was then restored. HDA-QD also induced mito-ROS, but to a substantially lower level than carboxyl-QD such that this change only appeared after 18h exposure at the top two doses and resulted in a smaller 1.5-fold increase compared with the controls (Figure 6C).

## Discussion

This study reports the variation in the genotoxic effects in TK6 cells exposed to QD as a function of surface chemistry and thus the charge of these NPs. Mechanisms resulting in potential genotoxicity were also explored by correlating the generation of chromosomal damage and point mutations with the presence of ROS and changes to mito-ROS.

Assessment of dispersion under experimental conditions revealed that the QD generally formed agglomerates in the presence of serum containing media with the following increasing order of average agglomerate size; carboxyl- > HDA- > amine-QD (Table 1). As QD come into contact with cells their properties and thus stability could be affected in terms of their ionic strength by formation of a protein corona and pH levels of their immediate environment. In general, NPs are internalised into the cell *via* endocytosis. During this process NPs are exposed to varying pH conditions (e.g. pH 7.4: culture medium; pH 5.5: late endosomes; and pH 4.5: lysosomes). Additionally, lysosomes may contain enzymes which can lead to the biodegradation of NPs and/or its protective coating, as reported by Sée *et al*. (36). In his study, Sée demonstrated that following endosomal uptake NPs or proteins conjugated or non-specifically bound to the cells were rapidly degraded by Cathepsin L, a low specificity protease, resulting in loss of functionality of the NPs. Therefore, the exposure of the surface of HDA-QD, in this study, to biological degrading and acidic environments may have resulted in grave effects on the particles soon following exposure, such as acid etching followed by release of metal ions. As the release of free ions has been strongly correlated with toxicity, the intraendosomal degradation of these QD could also have significant effects on cellular wellbeing (37).

To understand differences in QD surface chemistry on cellular uptake, ImageStream flow cytometry was used to quantify QD uptake in the form of mean fluorescence intensity within cells. ImageStream was selected because it enables high-throughput analysis of thousands of cells, with software algorithms that allow quantification of internalised NPs and not those attached to the outer cell surface, thereby enabling powerful quantitative uptake investigations (38). After 18h exposure, higher levels of cell-association were detected for carboxyl-QD compared to the other two QD at the same exposure concentration. This observation correlates with the literature where reports demonstrate carboxyl-QD are internalised by several different cell types (both human and non-human cells) to a greater extent than amine-QD.(22) This preferential uptake of carboxyl-QD may be due to the smaller agglomerates that form in cell media compared to the amine- or HDA-QD. Higher resolution analysis by confocal microscopy and TEM could detect only carboxyl-QD in the TK6 cells and these QD were largely confined to membrane bound vesicles suggesting that the primary route for uptake was endocytosis. No QD were identified inside the nucleus, but TEM only permits analysis of a limited cell number and thus the possibility of QD penetrating the nucleus cannot be disregarded.

TEM revealed that amine-QD formed large agglomerates that had the tendency to adhere to the cell membrane and confocal microscopy showed less uptake than the carboxyl-QD, explaining the low levels of uptake detected with the ImageStream (Figure 3). Positively charged NPs are thought to be more easily internalised by cells because of electrostatic attractions to the anionic cell membrane (39) however, in serum containing cell growth media, amine-QD have a negative charge (−5 mV; Table 1) and display substantial agglomeration, limiting cellular internalisation, perhaps because the large agglomerates hinder efficient endocytosis. Limited HDA-QD uptake was also noted, possibly due to the difference in the surface chemistry of these NPs compared with the highly charged carboxyl-QD that would influence the resultant protein corona that forms. Media is a complex environment containing a myriad of proteins, thus the protein corona that forms around the NPs will be acutely dependent upon their surface chemistry and charge. Thus, each of the QD assessed in this study would attract a different protein corona content given the variation in their surface chemistry (Table 1) that in turn will influence their capacity for cellular uptake (40,41). Another possible explanation could be related to the cellular exposure conditions. For example, the amine-QD form large agglomerates in culture media as determined in the DLS analysis and seen in confocal images. These large agglomerates could rapidly settle at the bottom of the flask. As TK6 cells grow in suspension and have a small surface area, this settling phenomenon may reduce overall QD exposure levels in these cells.

The cytotoxic effects of the QD on TK6 cells was assessed by calculating RPD which has been reported as one of the most reliable for cytotoxicity analysis (42); additionally, as it is not reliant on fluorescent reagents, there are no concerns surrounding interaction between the NP and the test system leading to false positive or negative results. HDA-QD induced significant cytotoxicity in TK6 cells at higher concentrations. Given HDA-QD demonstrated no measurable cellular uptake, it is possible that the observed effects are related to the dissolution of the QD that were seen at different pH levels representing different experimental environments resulting in the release of toxic cadmium heavy metal ions (43). Although cellular uptake was observed with two of the three QD, this did not always result in significant cytotoxicity and even though substantially higher uptake levels were noted following exposure to carboxyl-QD, this did not cause a significant reduction in cell viability.

Genotoxicity in the form of gross chromosomal damage and point mutagenicity was evaluated. TK6 suspension cells were more sensitive to HDA-QD exposure than to the carboxyl- or amine-QD. HDA-QD caused a dose-dependent increase in both chromosomal damage and a slight, albeit non-significant, increase in mutagenicity. Given the limited uptake, it is possible that this effect is caused by dissolution of the particles and release of free cadmium ions, and not damage induced directly by the QD particles themselves. From our dissolution studies, approximately 7% of the HDA-QD breakdown by Day 1 (24h post exposure) at neutral pH (Figure 2). Thus, given the size (4nm × 8nm) and particle concentration of the stock (6 x 10^15^ QD particles per L), if only 10% of the HDA-QD were to dissolve and transfer to the aqueous layer as Cd^2+^ there would be a concentration of 0.2 µg/ml in the 10nM dose. Interestingly, this concentration of Cd^2+^ has previously been demonstrated to cause both cytotoxicity and genotoxicity and thus correlates with the hypothesis of HDA-QD degradation causing the effects observed (44). Evaluating Cd^2+^ ions in parallel (e.g. by using CdCl_2_) can be useful in aiding discrimination between particle specific effects versus those caused by ion release following NP dissolution. Nonetheless, this approach also poses many technical problems in truly correlating between responses induced by the free metal ions from CdCl_2_ as compared with NP-derived ions. Firstly, the exposure concentration for CdCl_2_ is very difficult to define because not all quantum-dot associated ions will be leached and thus be responsible for the toxicity associated with the NPs. Secondly, the distribution of free ions from CdCl_2_ in the cell will be very different from the distribution of the QD NPs themselves. In the case of the NPs, some free Cd^2+^ ions may be present in the incubation media due to chemical equilibrium after synthesis. More importantly, most free Cd^2+^ ions will however be generated following cellular internalisation of the NPs, once they reside in the acidic environment of the endosomal or lysosomal compartments. There, they can reach high local concentrations that will affect cells in a very different manner than more homogenously distributed free Cd^2+^ ions (45). Consequently, measuring cellular free Cd^2+^ or cell-associated Cd^2+^ specifically released by the QD NPs would assist with defining the underlying mechanism in future studies. This may be achieved by technologies such as ICPMS, however discriminating between free Cd^2+^ ions and intact QD remains technically challenging and is not always possible to achieve.

Carboxyl-QD did induce dose-dependent increases in chromosomal damage and mutagenicity over the dose range assessed, but this did not achieve significance in either case and thus the damage induced was not notable. This was also true for the amine-QDs where no increase in either chromosomal damage or mutagenicity were noted. Although there are some reports on cytotoxicity and pro-inflammatory responses induced by QD, this is the first evaluation of QD genotoxicity based upon surface chemistry (46). The data presented therefore demonstrates that NP surface chemistry is not only important in determining the degree of cell uptake, but also the degree of material dissolution and the type of DNA damage that subsequently arises.

Countless studies have reported on the importance of oxidative damage as a mechanism for NP induced toxicity (22,47). Thus, to determine if oxidative stress was also responsible for the genotoxicity observed following QD exposure in TK6 cells the generation of ROS and mito-ROS were assessed. The data demonstrated that carboxyl- and HDA-QD were responsible for the production of ROS while no effects were noted in cells exposed to the amine-QD. Interestingly, there were clear differences in the type of ROS generated by each of these QD. Carboxyl-QD primarily induced mito-ROS formation after 4h exposures. HDA-QD also induced a significant increase in mito-ROS, but this was to a lesser extent than the carboxyl-QD, and only occurred at the highest concentrations applied for 18h. However, HDA-QD more predominantly induced the production of ROS at all the tested concentrations up to 10nM following 4h exposure. Although the increase in ROS levels in cells exposed to HDA-QDs were accompanied with genotoxic effects, this was not the case with carboxyl-QD treatments, which suggests that mito-ROS is not as potent at inducing genotoxicity. It is also possible, that scavenging of mito-ROS is more efficient that cellular ROS, but further exploration would be required to provide the evidence to support this theory. These differences could also be due to possible variations in the stability of these NPs in the intracellular environment where exposure to different pH conditions could result in particle degradation (as we observed here) resulting in differences in their interaction with intracellular molecules (48). This is particularly pertinent as cadmium induces aneuploidy *via* alterations in DNA-methylation status, and thus it would be interesting to determine if aberrant DNA-methylation status is also associated with cellular uptake of QD (49).

Taken together, the data demonstrate that the carboxyl-QD, which were internalised to the greatest extent, induced a significant increase in mito-ROS that was coupled to a slight reduction in cell viability, but limited genotoxicity. HDA-QD, did not demonstrate notable levels of uptake, but did cause an intermediate increase in mito-ROS. HDA-QD also induced substantial cytotoxicity and chromosomal damage, which may however be due to particle dissolution. Given that the cytotoxicity and genotoxicity induced by HDA-QD occur at lower concentrations that those responsible for elevating mito-ROS, it would strongly suggest that there are other mechanisms of action of importance. Consequently, the cyto- and genotoxic effects of QD could be inter-linked to the intracellular concentration of the QD, the presence of additional factors that could pose cellular stress such as QD degradation, and the surface chemistry of the NPs that result in NP-specific genotoxic profiles.

## Conclusions

The present work focuses on comparing variations in the nature of cellular interactions between human cells and QD, based on differences in QD surface chemistry. All QD cores were of a similar size and composition, however, the colloidal stability and degree of agglomeration was found to differ substantially between the QD according to surface chemistry. Positively charged amine-QD was found to agglomerate most extensively, whereas the negatively charged carboxyl-QD agglomerated to a much lesser extent. These differences in agglomeration and colloidal stability led to significant variation in the resultant cellular interactions. The carboxyl-QD demonstrated the most pronounced uptake levels. The high internalisation of carboxyl-QD also resulted in the greatest induction of mito-ROS. However, despite this, genotoxicity induced by carboxyl-QD was minimal. In contrast, the greatest genotoxicity and cytotoxicity was induced by HDA-QD, which were associated with low cell uptake and limited induction of oxidative stress. Considering the low particle uptake noted and the particle breakdown observed at varying pH levels, it is possible that the observed toxicity is mainly induced by free cadmium ion release. Consequently, although surface chemistry is an important parameter governing dissolution and cellular uptake, the stability and intrinsic toxicity of the nanomaterial itself together represent the key determinants for induced genotoxicity.
